# Stereotactic radiosurgery for 1–10 brain metastases to avoid whole-brain radiotherapy: Results of the CYBER-SPACE randomized phase 2 trial

**DOI:** 10.1093/neuonc/noae201

**Published:** 2024-09-28

**Authors:** Rami A El Shafie, Denise Bernhardt, Thomas Welzel, Annabella Schiele, Daniela Schmitt, Paul Thalmann, Sinem Erdem, Angela Paul, Simon Höne, Kristin Lang, Laila König, Fabian Weykamp, Sebastian Adeberg, Adriane Lentz-Hommertgen, Cornelia Jäkel, Farastuk Bozorgmehr, Ursula Nestle, Michael Thomas, Anja Sander, Meinhard Kieser, Jürgen Debus, Stefan Rieken

**Affiliations:** Department of Radiation Oncology, University Hospital Heidelberg, Heidelberg, Germany; Heidelberg Institute of Radiation Oncology (HIRO), Heidelberg, Germany; National Center for Tumor Diseases (NCT), Heidelberg, Germany; Department of Radiation Oncology, University Medical Center Göttingen, Göttingen, Germany; Department of Radiation Oncology, University Hospital Heidelberg, Heidelberg, Germany; Heidelberg Institute of Radiation Oncology (HIRO), Heidelberg, Germany; National Center for Tumor Diseases (NCT), Heidelberg, Germany; Department of Radiation Oncology, School of Medicine and Klinikum rechts der Isar, Technical University of Munich (TUM), Munich, Germany; Department of Radiation Oncology, University Hospital Heidelberg, Heidelberg, Germany; Heidelberg Institute of Radiation Oncology (HIRO), Heidelberg, Germany; National Center for Tumor Diseases (NCT), Heidelberg, Germany; Department of Radiation Oncology, University Hospital Heidelberg, Heidelberg, Germany; Heidelberg Institute of Radiation Oncology (HIRO), Heidelberg, Germany; National Center for Tumor Diseases (NCT), Heidelberg, Germany; Department of Radiation Oncology, University Hospital Heidelberg, Heidelberg, Germany; Heidelberg Institute of Radiation Oncology (HIRO), Heidelberg, Germany; National Center for Tumor Diseases (NCT), Heidelberg, Germany; Department of Radiation Oncology, University Medical Center Göttingen, Göttingen, Germany; Institute of Medical Biometry, University of Heidelberg, Heidelberg, Germany; Department of Radiation Oncology, University Hospital Heidelberg, Heidelberg, Germany; Heidelberg Institute of Radiation Oncology (HIRO), Heidelberg, Germany; National Center for Tumor Diseases (NCT), Heidelberg, Germany; Department of Radiation Oncology, University Hospital Heidelberg, Heidelberg, Germany; Heidelberg Institute of Radiation Oncology (HIRO), Heidelberg, Germany; National Center for Tumor Diseases (NCT), Heidelberg, Germany; Department of Radiation Oncology, University Hospital Heidelberg, Heidelberg, Germany; Heidelberg Institute of Radiation Oncology (HIRO), Heidelberg, Germany; National Center for Tumor Diseases (NCT), Heidelberg, Germany; Department of Radiation Oncology, University Hospital Heidelberg, Heidelberg, Germany; Heidelberg Institute of Radiation Oncology (HIRO), Heidelberg, Germany; National Center for Tumor Diseases (NCT), Heidelberg, Germany; Department of Radiation Oncology, University Hospital Heidelberg, Heidelberg, Germany; Heidelberg Institute of Radiation Oncology (HIRO), Heidelberg, Germany; National Center for Tumor Diseases (NCT), Heidelberg, Germany; Department of Radiation Oncology, University Hospital Heidelberg, Heidelberg, Germany; Heidelberg Institute of Radiation Oncology (HIRO), Heidelberg, Germany; National Center for Tumor Diseases (NCT), Heidelberg, Germany; Department of Radiation Oncology, University Hospital Heidelberg, Heidelberg, Germany; Heidelberg Institute of Radiation Oncology (HIRO), Heidelberg, Germany; National Center for Tumor Diseases (NCT), Heidelberg, Germany; Department of Radiation Oncology, University Hospital Marburg, Marburg, Germany; Department of Radiation Oncology, University Hospital Heidelberg, Heidelberg, Germany; Heidelberg Institute of Radiation Oncology (HIRO), Heidelberg, Germany; National Center for Tumor Diseases (NCT), Heidelberg, Germany; Department of Radiation Oncology, University Hospital Heidelberg, Heidelberg, Germany; Heidelberg Institute of Radiation Oncology (HIRO), Heidelberg, Germany; National Center for Tumor Diseases (NCT), Heidelberg, Germany; National Center for Tumor Diseases (NCT), Heidelberg, Germany; Department of Thoracic Oncology, Thoraxklinik, Heidelberg University, Heidelberg, Germany; Translational Lung Research Centre Heidelberg (TLRC-H), German Centre for Lung Research (DZL), Heidelberg, Germany; Department of Radiation Oncology, Freiburg University Medical Center, Freiburg, Germany; Department of Radiation Oncology, Kliniken Maria Hilf, Moenchengladbach, Germany; Department of Radiation Oncology, University Hospital Freiburg, Freiburg, Germany; National Center for Tumor Diseases (NCT), Heidelberg, Germany; Department of Thoracic Oncology, Thoraxklinik, Heidelberg University, Heidelberg, Germany; Translational Lung Research Centre Heidelberg (TLRC-H), German Centre for Lung Research (DZL), Heidelberg, Germany; Institute of Medical Biometry, University of Heidelberg, Heidelberg, Germany; Institute of Medical Biometry, University of Heidelberg, Heidelberg, Germany; Department of Radiation Oncology, University Hospital Heidelberg, Heidelberg, Germany; Heidelberg Institute of Radiation Oncology (HIRO), Heidelberg, Germany; National Center for Tumor Diseases (NCT), Heidelberg, Germany; Heidelberg Ion-Beam Therapy Center (HIT), Heidelberg, Germany (J.D.); German Cancer Consortium (DKTK), Heidelberg, Germany; Clinical Cooperation Unit Radiation Oncology, German Cancer Research Center (DKFZ), Heidelberg, Germany; Department of Radiation Oncology, University Hospital Heidelberg, Heidelberg, Germany; Heidelberg Institute of Radiation Oncology (HIRO), Heidelberg, Germany; National Center for Tumor Diseases (NCT), Heidelberg, Germany; Department of Radiation Oncology, University Medical Center Göttingen, Göttingen, Germany

**Keywords:** MRI monitoring, multiple brain metastases, stereotactic radiosurgery (SRS), SPACE sequence, whole-brain radiotherapy (WBRT)

## Abstract

**Background:**

Stereotactic radiosurgery (SRS) is an emerging alternative to whole-brain radiotherapy (WBRT) for treating multiple brain metastases (BM), reducing toxicity, and improving tumor control. The CYBER-SPACE trial compared SRS based on either SPACE or MPRAGE MRI sequence for avoiding or delaying WBRT in patients with 1–10 BM.

**Methods:**

Patients with 1–10 untreated BM were randomized 1:1 to receive SRS of all lesions based on either SPACE or MPRAGE MRI sequences. If subsequently new BM occurred, SRS was repeated. WBRT was indicated upon occurrence of >10 new BM, leptomeningeal disease, or exhausted SRS-radiotolerance. The primary outcome was freedom from WBRT indication (WBRTi). Secondary outcomes included overall survival (OS), safety, and quality of life.

**Results:**

A total of 202 patients were randomized; SPACE *n* = 99, MPRAGE *n* = 103. Twelve-month WBRTi-free survival was 77.1% (95% CI: 69.5%–83.1%) overall, 78.5% (95% CI: 66.7%–86.5%) for SPACE, and 76.0% (95% CI: 65.2%–83.9%) for MPRAGE (hazard ratio [HR] = 0.84, 95% CI: 0.43–1.63, *P* = .590). Patients with 5–10 BM had shorter WBRTi-free survival (HR = 3.13, 95% CI: 1.53–6.40, *P* = .002). Median OS was 13.1 months overall, 10.5 months for SPACE, and 15.2 months for MPRAGE (HR = 1.10, 95% CI: 0.78–1.56, *P* = .585). Neurologic death rate was 10.1%. Predictors for longer OS included Karnofsky Performance Status >80% (HR = 0.51, 95% CI: 0.33–0.77, *P* = .002) and concurrent immunotherapy (HR = 0.34, 95% CI: 0.23–0.52, *P* < .001).

**Conclusions:**

The more sensitive SPACE sequence did not improve outcomes over MPRAGE. SRS with thorough monitoring and immediate re-treatment for new lesions decreases the need for WBRT and achieves low neurologic death rates. SRS should be considered a favorable alternative to WBRT for patients with 1–10 BM.

**Importance of the Study**

The CYBER-SPACE phase 2 trial is the first randomized trial to evaluate the use of stereotactic radiosurgery (SRS) in patients with 1–10 brain metastases (BM) with the aim of avoiding or delaying the need for whole-brain radiotherapy (WBRT) and its relevant toxicities. The use of the more sensitive SPACE MRI sequence for treatment and follow-up did not yield a clinical advantage over MPRAGE sequence in randomized comparison. SRS, followed by rigorous MRI monitoring and timely re-treatment for new lesions, achieved high freedom from WBRT indication (by observer-independent criteria) of 77.1% at 12 months and low neurologic death rates of 10.1%. Simultaneous combination of SRS with standard-of-care immunotherapy or targeted therapy did not increase toxicity and improved overall survival. These results support SRS as a favorable, less toxic alternative to WBRT in the era of modern systemic treatment, and highlight the importance of close monitoring and active management of new lesions.

**Key Points**

- Repeated stereotactic radiosurgery (SRS) for multiple brain metastases avoids whole-brain radiotherapy (WBRT) and neurologic death.
- The more sensitive SPACE MRI sequence did not improve outcomes over MPRAGE sequence.
- SRS with concurrent immuno-/targeted therapies is well tolerated and associated with favorable overall survival.

Approximately one-third of adult cancer patients develop brain metastases (BM), leading to significant neurologic morbidity and mortality. The incidence of BM is increasing, attributed to more sensitive cranial imaging and extended survival with advanced systemic but not necessarily cerebrally effective treatments.^1^

During the past decades, local treatment options for BM have greatly evolved. Several randomized trials have established stereotactic radiosurgery (SRS) for optimizing local control in limited BM (1–4 lesions). Although the addition of whole-brain radiotherapy (WBRT) to surgery or SRS reduces the risk of distant intracranial recurrence, it does not improve overall survival (OS) and is linked with significant neurocognitive toxicity, impacting quality of life (QoL).^2–6^

Consequently, more recent studies have sought to avoid WBRT in favor of SRS for multiple BM. The pivotal study by Yamamoto et al. demonstrated that SRS without WBRT for patients with 5–10 BM was non-inferior in OS compared to patients with 2–4 metastases.^7^ SRS could thus be a suitable alternative even for patients with a higher number of BM, challenging the automatic recommendation of WBRT in such cases. However, post-SRS distant intracranial recurrences affect more than half of the patients and require close monitoring and effective salvage treatments.^7^

Advancements in radiation oncology, like robotic radiosurgery and non-coplanar single-isocenter multiple metastases techniques, have expanded the mainstream adoption of SRS for treating multiple lesions efficiently.^8–11^ Enhanced pretreatment MRI, particularly using 3 Tesla scanners and high-resolution, three-dimensional (3D) sequences, has improved lesion detection and treatment planning.^12^ Specifically, the SPACE sequence (sampling perfection with application-optimized contrasts by using different flip angle evolutions) has previously shown superior sensitivity and specificity in BM detection, compared to the commonly used MPRAGE sequence (magnetization-prepared rapid gradient-echo).^13,14^

In our trial, we therefore aimed to determine, if SRS of multiple BM on the basis of the more sensitive SPACE rather than MPRAGE MRI, followed by rigorous MRI monitoring and repeated SRS for new lesions based on the respective MRI sequence, was superior in avoiding or delaying the need for WBRT. Patients on trial would have an indication for salvage WBRT (WBRTi), if a degree of intracranial dissemination not amenable to further SRS was reached during follow-up. Freedom from WBRTi, defined by robust, observer-independent criteria, was therefore chosen as a clinically meaningful primary outcome.

## Methods

### Study Design and Participants

CYBER-SPACE (NCT03303365) was a prospective randomized phase 2 single-center trial. We included adult patients (≥18 years of age) from a tertiary cancer center with up to 10 distinct, untreated BM of solid tumors and adequate clinical performance (Karnofsky Performance Score [KPS] ≥70). To avoid potential imbalances in the number of lesions at inclusion, lesion count to determine eligibility was done on the more sensitive SPACE sequence in both arms and followed a standardized, central MRI assessment workflow, as illustrated in Supplementary Figure S1. Large metastases (>3 cm) could be included if deemed eligible for hypofractionated radiosurgery by the investigator. Though not defined explicitly, lesions >4 cm were generally not considered eligible and referred to neurosurgery instead, if clinically feasible. Patients could have active extracranial disease and could be treated with systemic agents while on the trial. Key exclusion criteria were small-cell lung cancer (SCLC) as primary histology, known contraindication for MR imaging, radiographic evidence of leptomeningeal disease (LMD), as well as previous radiotherapy to the brain. No systematic screening for LMD by cerebrospinal fluid (CSF) cytology was done during screening or follow-up. The complete eligibility criteria are listed in the trial protocol (Supplementary Appendix). Ethics approval for the trial was granted by the institutional review board on September 21, 2017 (#S-448/2017). All patients were required to provide written informed consent.

### Randomization and Masking

Patients were randomly assigned (1:1) to receive SRS for all BM visible on the respective MRI sequence (SPACE or MPRAGE), depending on study arm, as well as follow-up and re-treatment for new BM based on the respective MRI sequence. Randomization used a permuted block procedure (block lengths 4 and 2) via a centralized web-based tool (www.randomizer.at). Randomization was stratified by histology (lung cancer vs radioresistent histology (melanoma, renal cell carcinoma, sarcoma) vs other) and the largest diameter of the largest metastasis (<3 cm vs ≥3 cm). Patients and clinicians were not masked to treatment assignment. Statisticians were masked until database closure.

### Procedures

Pre-treatment MRI was performed at a 3.0 Tesla scanner (MAGNETOM Skyra, Siemens) using a standardized imaging protocol as described previously.^15^ The protocol included Gadolinium-based (0.1 mmol/kg) MPRAGE and SPACE sequences (always performed in that order) with a slice thickness of 1 mm and multiplanar reconstruction, as well as a T2-weighted sequence. Time requirement for MPRAGE was approximately 4 minutes, and 5 minutes for SPACE. All MRI scans (baseline and follow-up) were reviewed centrally by the study radiologist via a standardized workflow that considered only MPRAGE-visible lesions in the MPRAGE arm and SPACE-visible lesions in the SPACE arm for initial treatment and re-treatments (Supplementary Figure S1).

The SPACE sequence is a 3D fast spin echo sequence that combines high-contrast enhancement with a high spatial resolution and multiplanar reconstruction.^12^ This sequence was found to have improved sensitivity and specificity in the detection of BM, compared to a 3D gradient echo sequence such as MPRAGE. This difference was accentuated in the detection of small lesions <5 mm in size.^13,14,16^

For treatment planning, the respective MRI was co-registered to a planning-computed tomography scan with 1 mm slice thickness and the contrast-enhancing lesion on MRI was defined as gross tumor volume. A 1 mm isotropic margin was added for planning target volume (PTV). Frameless robotic SRS was performed at a CyberKnife M6 (Accuray Inc.) system with intrafractional motion tracking using stereoscopic X-ray systems. Dose prescription to the isodose conformally surrounding at least 98% of the PTV (margin dose) was as follows, and no dose modifications/reductions (eg, for concomitant systemic treatment) were allowed in the trial:

- 1 × 20 Gy to 70% isodose (lesions <2 cm maximum diameter).
- 1 × 18 Gy to the 70% isodose (lesions 2–3 cm maximum diameter).
- 6 × 5 Gy to the 70% isodose (lesions >3 cm maximum diameter).

### Assessments

Baseline assessments were completed before initial treatment and consisted of medical history review, physical and neurological exams, performance status evaluation, and high-resolution standardized MRI. Quality of life was assessed using the European Organization for Research and Treatment of Cancer QLQ-C15-PAL and the BN20 modules. Baseline neurocognitive testing encompassed the Hopkins Verbal Learning Test-Revised (HVLT-R) total and delayed recall, as well as a validated tablet-based test battery by CANTAB (Cambridge Neuropsychological Test Automated Battery, www.cambridgecognition.com [Cambridge Cognition]).

Following initial treatment, patients were followed up in 3-monthly intervals, including the same standardized high-sensitivity MRI protocol used initially. Lesion detection for re-treatment during follow-up was based on SPACE or MPRAGE depending on study arm, as explained above and in Supplementary Figure S1. If during follow-up new BM occurred, per-protocol treatment using SRS was repeated for those new lesions, as long as re-treatment preserved the required radiotolerance for performing salvage WBRT later on, if necessary. This was defined as a dose reserve for healthy brain and other organs at risk of at least 37.5 Gy (equivalent dose in 2Gy [EQD2] for α/β = 2, see study protocol section 7.2 in Supplementary Appendix). Additionally, clinical examination, QoL assessment, and neurocognitive testing were repeated at each follow-up examination.

### Outcomes

The primary endpoint was cerebral progression with ineligibility for further SRS (iffS, defined as the simultaneous (ie, in a single follow-up scan) new occurrence or progression of >10 BM, LMD by radiographic evidence or exhausted SRS-radiotolerance) as a robust, observer-independent surrogate endpoint for the indication for WBRT (WBRTi). The corresponding primary analysis was freedom from WBRTi. A decision algorithm for assessing the primary endpoint is illustrated in Supplementary Figure S2. Lesion count for assessment of WBRTi was done on the more sensitive SPACE sequence for all patients, irrespective of study arm, to avoid imbalances due to the different sensitivities of the MRI sequences. The rate of actually performed salvage WBRT treatments was described as a secondary outcome. Other key secondary outcomes were OS and safety. Further prespecified outcomes were QoL and neurocognitive function, which will be reported separately.

### Statistical Analysis

The aim of this trial was to determine, if treatment on the basis of SPACE MRI sequence provided superior freedom from WBRTi than treatment on the basis of MPRAGE MRI sequence. Thus, the rate of patients reaching ineligibility for further SRS (iffS) in the SPACE arm, defined as pA, would be smaller than the respective rate of patients in the MPRAGE arm, pB. Based on previously published studies, it was assumed that pA amounts to 6%, while pB is as high as 20%.^7,13,17^ Under these assumptions, *n* = 90 patients per group were required in order to detect a difference between the 2 groups at a 2-sided significance level of α = 0.05 with a probability of 1−β = 0.80 using a chi-squared test. Taking a dropout rate of 10% into account, the total sample size for the trial thus amounted to *n* = 200 patients.

IffS and correspondingly freedom from WBRTi were to be evaluated 12 months after initial SRS. To allow for a more robust and comprehensive analysis, freedom from WBRTi was also analyzed as a time-to-event endpoint, considering all available follow-up data up to 24 months. Patients without an event were censored at 24 months from initial SRS. Median follow-up was calculated using the method of reverse Kaplan–Meier.^18^ Kaplan–Meier plots and *P* values of descriptive Log-rank tests were given. Death of all causes as a competing event was considered using a subdistribution hazard model. Cumulative incidence function plots and *P* values of Gray-tests were given. A Cox proportional hazards model controlling for multiple covariates selected via a predefined strategy and considering death as a competing event was calculated. Variable selection was conducted by fitting models, which only included the treatment group and one variable of interest. Variables with a *P* value < .1 in this initial step were included in the multivariable model. When only one category of a categorical variable had a *P* value < .1, all possible categories were included. If applicable, every point estimate was reported with a corresponding 95% confidence interval.

For OS, similar analyses were conducted, employing Kaplan–Meier plots, descriptive Log-rank tests, and Cox proportional hazard models. For OS, all available follow-up data were considered, exceeding 24 months, if available.

Treatment-related adverse events (TRAE) were analyzed descriptively. To compare TRAE incidence between subgroups, a post hoc analysis of exposure-adjusted incidence rates was done by normalizing the average incidence rate per patient by the time on trial.

Efficacy analyses followed the intention-to-treat principle. The modified intention-to-treat (mITT) set included all randomized patients in their allocated group who started the study treatment, regardless of whether they completed 24 months on study or exited early (eg, due to death). The per-protocol (PP) set consisted of mITT patients without major protocol deviations, which were judged case by case. The safety set included mITT patients analyzed according to the treatment they received. As there were no major protocol violations and all patients received their allocated treatment, the mITT, PP, and safety sets are identical in this trial.

All analyses were done in either SAS version 9.4 or R version 4.2.2 and higher. Database closure was done on February 2, 2023.

## Results

### Study Patients

**Figure 1.**
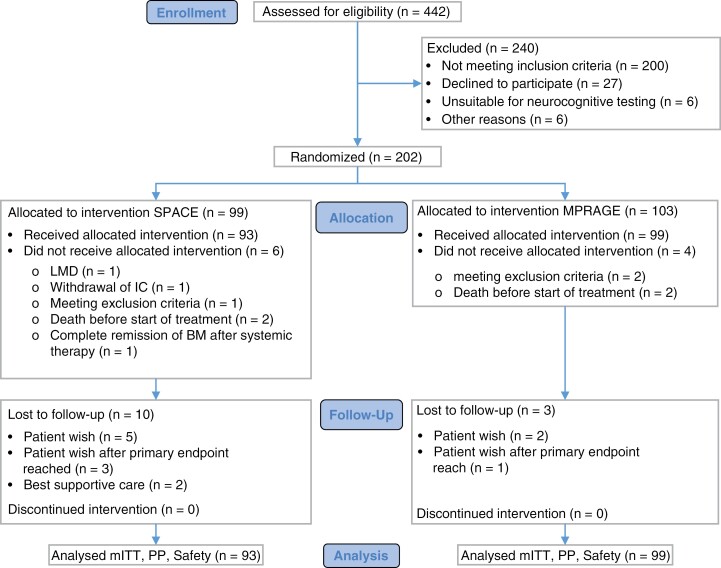
CONSORT flow chart of enrolled patients. BM = brain metastases; IC = informed consent; LMD = leptomeningeal disease.

Between January 2018 and June 2020, 202 patients were randomly assigned to receive SRS of all visible BM according to either SPACE (*n* = 99) or MPRAGE (*n* = 103) MRI sequence. Six patients in the SPACE arm and 4 patients in the MPRAGE arm were found ineligible after randomization and were excluded before receiving study treatment (Figure 1, Supplementary Table S1). Ten patients (SPACE) and 3 patients (MPRAGE) prematurely discontinued follow-up but were analyzed in the mITT set. Of those, 3 patients (SPACE) and 1 patient (MPRAGE) reached WBRTi before discontinuing follow-up. The mITT data set, per-protocol data set and safety data set encompassed 93 SPACE patients and 99 MPRAGE patients (Figure 1).

Baseline characteristics were well balanced between the study arms (Table 1). Median patient age in the overall cohort was 62.5 (Q1–Q3: 55.0–68.0) years. The predominant primary histology was non–small-cell lung cancer (NSCLC) in 63.0% of cases, followed by melanoma (16.1%) and breast cancer (9.9%). Baseline number of BM was similar between arms. In the combined cohort, it was 1 BM in 24.5% (*n* = 47), 2–4 BM in 47.4% (*n* = 91), and 5–10 BM in 28.1% (*n* = 54) of patients. Extracerebral disease was controlled in 27.6% (*n* = 53) and not controlled in 72.4% (*n* = 139) of patients. Over the course of the trial and with repeated treatments, 17.2% (*n* = 33) of patients were treated at 1 BM, 41.7% (*n* = 80) at 2–4 BM, 28.6% (*n* = 55) at 5–10 BM, and 12.5% (*n* = 24) at >10 lesions. Number of total SRS courses was similar between arms, with 56.3% (*n* = 108) of the combined cohort receiving 1 course, 20.8% (*n* = 40) receiving 2 courses, and 22.2% (*n* = 44) receiving 3 or more courses (Table 2).

**Table 1.** Baseline Characteristics in the Study Collective

Histology	SPACE (*n* = 93)	MPRAGE *(n* = 99)	Total (*n* = 192)	*P* value
NSCLC	59 (63.4%)	62 (62.6%)	121 (63.0%)	0.99^Chi-sq^
Melanoma	15 (16.1%)	16 (16.2%)	31 (16.1%)	
Breast	8 (8.6%)	11 (11.1%)	19 (9.9%)	
Renal cell	2 (2.2%)	2 (2.0%)	4 (2.1%)	
GI tract	1 (1.1%)	1 (1.0%)	2 (1.0%)	
Other	8 (8.6%)	7 (7.1%)	15 (7.8%)	
**Age, years**				
Mean (SD)	61.6 (11.6)	61.9 (9.6)	61.8 (10.6)	0.86^*t* test^
Median (Q1–Q3)	62.0 (54.0–69.0)	63.0 (56.0–68.0)	62.5 (55.0–68.0)	
**Sex**				
Female	48 (51.6%)	44 (44.4%)	92 (47.9%)	0.32^Chi-sq^
Male	45 (48.4%)	55 (55.6%)	100 (52.1%)	
**Extracerebral disease**				
Controlled	25 (26.9%)	28 (28.3%)	53 (27.6%)	0.63^Chi-sq^
Not controlled	68 (73.1%)	71 (71.7%)	139 (72.4%)	
**Karnofsky Performance Status (≥90 vs ≤80)**				
≤80	41 (44.1%)	55 (55.6%)	96 (50.0%)	0.11^Chi-sq^
≥90	52 (55.9%)	44 (44.4%)	96 (50.0%)	
**Number of lesions at baseline**				
1	23 (24.7%)	24 (24.2%)	47 (24.5%)	0.34^Chi-sq^
2–4	44 (47.3%)	47 (47.5%)	91 (47.4%)	
5–10	26 (28.0%)	28 (28.3%)	54 (28.1%)	
**Maximum diameter of the largest metastasis (mm)**				
Mean (SD)	13.6 (8.0)	11.9 (7.1)	12.7 (7.6)	0.12^*t* test^
Median (Q1–Q3)	12.0 (7.0–18.0)	10.0 (6.0–17.0)	11.0 (6.0–17.0)	
Min, Max	2.0, 40.0	3.0, 35.0	2.0, 40.0	
**RPA class**				
1	10 (10.8%)	3 (3.0%)	13 (6.8%)	0.03^Chi-sq^
2	83 (89.2%)	96 (97.0%)	179 (93.2%)	
**GPA score**				
3.5–4.0	2 (2.2%)	1 (1.0%)	3 (1.5%)	0.62^Chi-sq^
3.0	9 (9.7%)	6 (6.1%)	15 (7.8%)	
1.5–2.5	60 (64.5%)	63 (63.6%)	123 (64.1%)	
0–1	22 (23.6%)	29 (29.2%)	51 (26.6%)	
**Concomitant therapy**				
Chemotherapy	80 (58.8%)	87 (52.4%)	167 (55.3%)	0.573^Chi-sq^
Palliative radiotherapy	37 (27.2%)	62 (37.3%)	99 (32.8%)	
Immunotherapy	47 (50.5%)	46 (46.5%)	93 (48.4%)	
Targeted therapy	31 (33.3%)	38 (38.4%)	69 (35.9%)	
Surgery	11 (8.1%)	9 (5.4%)	20 (6.6%)	
Chemoradiation	2 (1.5%)	1 (0.6%)	3 (1.0%)	
Other^*^	6 (4.4%)	7 (4.2%)	13 (4.3%)	
None	8 (8.6%)	10 (10.1%)	18 (9.4%)	
**Concomitant therapy combinations**				
Monotherapy	51 (54.8%)	50 (50.5%)	101 (52.6%)	0.573^Chi-sq^
Combined therapies	34 (36.6%)	39 (39.4%)	73 (38.0%)	
No therapy	8 (8.6%)	10 (10.1%)	18 (9.4%)	

Data are numbers (%), unless otherwise speciﬁed.

GI = gastrointestinal; GPA = graded prognostic assessment; RPA = recursive partitioning analysis.

^*^Other concomitant therapies: hormonal therapy, experimental vaccines, osteoprotective therapy.

**Table 2.** Treatment and Outcome Parameters Over the Course of the Trial

	SPACE (*n* = 93)	MPRAGE (*n* = 99)	Total (*n* = 192)
**Lesions treated with**			
20 Gy	463 (88.9%)	445 (88.3%)	908 (88.6%)
18 Gy	35 (6.7%)	38 (7.5%)	73 (7.1%)
6 × 5 Gy	23 (4.4%)	21 (4.2%)	44 (4.3%)
**SRS courses per patient before reaching WBRTi (including re-treatment of new lesions during follow-up)**			
1	55 (59.1%)	53 (53.5%)	108 (56.3%)
2	18 (19.4%)	22 (22.2%)	40 (20.8%)
3	10 (10.8%)	9 (9.1%)	19 (9.9%)
≥4	10 (10.8%)	15 (15.2%)	25 (13.0%)
**Total number of treated lesions before reaching WBRTi**			
1	16 (17.2%)	17 (17.2%)	33 (17.2%)
2–4	36 (38.7%)	44 (44.4%)	80 (41.7%)
5–10	28 (30.1%)	27 (27.3%)	55 (28.6%)
>10	13 (14.0%)	11 (11.1%)	24 (12.5%)
Median (Q1–Q3)	4 (2–8)	3 (2–7)	4 (2–8)
Mean (SD)	5.6 (5.1)	5.1 (4.6)	5.3 (4.8)
**Reasons for WBRTi**			
>10 new BM	11 (64.7%)	15 (65.2%)	26 (65.0%)
LMD	4 (23.5%)	5 (21.7%)	9 (22.5%)
LMD and >10 new BM	2 (11.8%)	3 (13.0%)	5 (12.5%)
Exhausted SRS-radiotolerance	0 (0%)	0 (0%)	0 (0%)
**Performed salvage therapy for WBRTi**			
WBRT	13 (14.0%)	14 (14.1%)	27 (14.1%)
WBRT + systemic treatment	0 (0.0%)	2 (2.0%)	2 (1.0%)
Systemic treatment	1 (1.1%)	2 (2.0%)	3 (1.6%)
Best supportive care	3 (3.2%)	3 (3.0%)	6 (3.1%)
SRS	0 (0.0%)	1 (1.0%)	1 (0.5%)
Any CNS-directed intervention	14 (15.1%)	19 (19.2%)	33 (17.2%)
**Died**			
Due to BM	6 (6.5%)	8 (8.1%)	14 (7.3%)
Due to extracranial disease	49 (52.7%)	45 (45.5%)	94 (49.0%)
Unknown	13 (14.0%)	17 (17.2%)	30 (15.6%)

Data are numbers (%), unless otherwise speciﬁed. BM = brain metastases; Gy = Gray; LMD = leptomeningeal disease; SRS = sterotactic radiosurgery; WBRT = whole-brain radiotherapy; WBRTi = indication for whole-brain radiotherapy.

### Freedom From Whole-Brain Radiotherapy Indication

Median follow-up was 23.2 [Q1–Q3: 8.0–24.0] months. At 12 months from initial SRS, 37 patients (19.3%) in the overall cohort reached WBRTi according to per-protocol definition, 16 (17.2%) in the SPACE arm, and 21 (21.2%) in the MPRAGE arm. By 24 months, these rates were 40 patients (20.8%) overall, 17 (18.3%) in the SPACE arm, and 23 (23.2%) in the MPRAGE arm.

**Figure 2.**
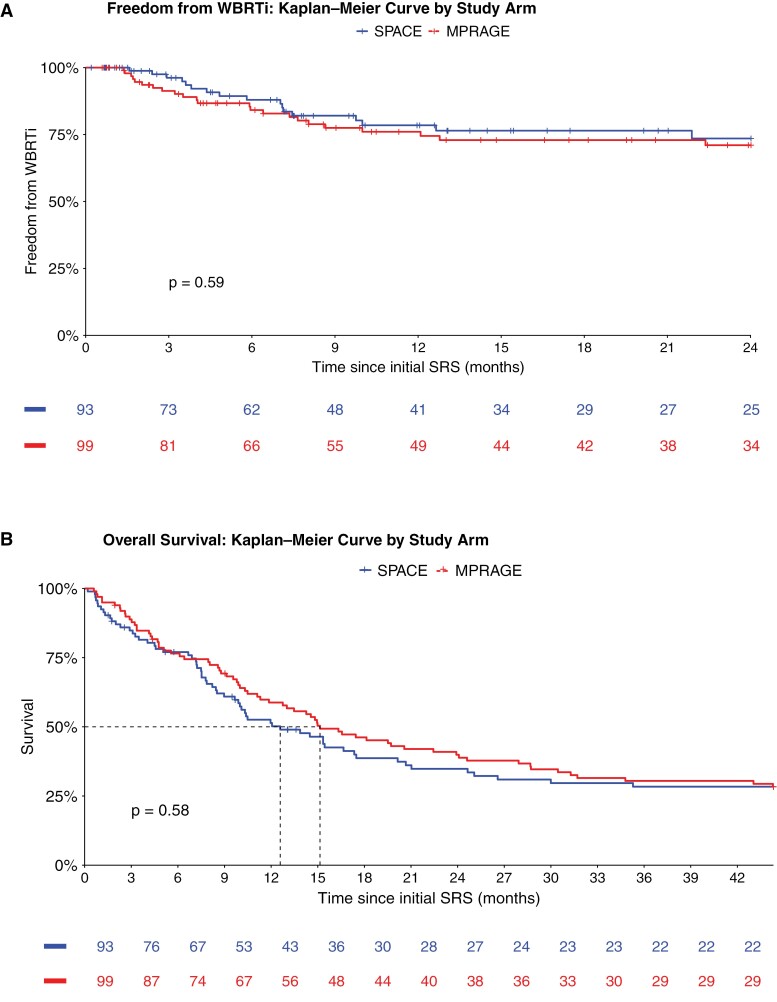
(A) Kaplan–Meier curves of freedom from whole-brain radiotherapy indication (WBRTi). (B) Kaplan–Meier curves of overall survival.

WBRTi-free survival at 12 months by Kaplan–Meier estimate was 77.1% (95% CI: 69.5%–83.1%) in the overall cohort, 78.5% (95% CI: 66.7%–86.5%) in the SPACE arm, and 76.0% (95% CI: 65.2%–83.9%) in the MPRAGE arm. At 24 months, it was 72.2% (95% CI: 63.7%–79.0%) overall, 73.5% (95% CI: 60.1%–83.0%) in the SPACE arm, and 71.0% (95% CI: 59.3%–79.9%) in the MPRAGE arm. Median WBRTi-free survival was not reached in either arm, with no significant difference between arms (hazard ratio [HR] 0.84 [95% CI: 0.43–1.63], log-rank *P* = .590; Figure 2A).

**Figure 3.**
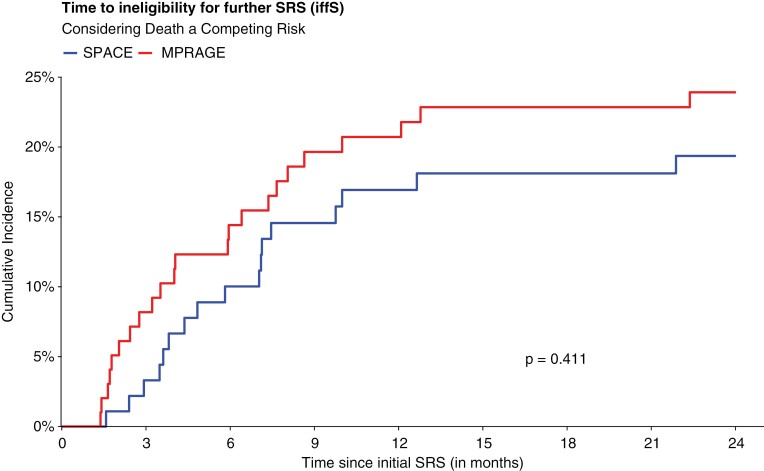
Cumulative incidence of cerebral progression with ineligibility for further SRS (iffS) in both study arms, considering death as a competing risk.

Competing risk analysis showed WBRTi rates at 24 months of 21.7% (95% CI: 16.1%–27.9%) overall, 19.4% (95% CI: 11.8%–28.3%) in the SPACE arm, and 23.9% (95% CI: 15.9%–32.9%) in the MPRAGE arm (Figure 3).

#### Salvage therapy upon WBRTi

Of the 40 patients reaching WBRTi, reasons were radiographic LMD in 9 (22.5%) cases, >10 new BM in 26 cases (65%), and a combination of both in 5 cases (12.5%). Following current guidelines, CSF cytology was individually indicated for confirmation or further workup of LMD (eg, molecular diagnostics) in 3/14 patients (21.4%) with radiographic LMD. Salvage treatment for WBRTi was discussed in a multidisciplinary panel, considering individual case history, extracranial tumor status, and patient wish. Of patients with WBRTi, 27 (67.5%) actually received WBRT, 2 (5.0%) received WBRT with additional CNS-directed systemic treatment, 3 (7.5%) received only systemic treatment, 6 (15.0%) received best supportive care, and 1 (2.5%) received additional SRS (Table 2). Considering performed salvage treatments, actual freedom from WBRT at 24 months was 84.9% (163 patients).

### Overall Survival

Median overall survival was 13.1 months (86 deaths, Q1–Q3: 5.2–44.0) in both groups combined, 10.5 months (40 deaths, Q1–Q3: 5.1–30.4) in the SPACE arm, and 15.2 months (47 deaths, Q1–Q3: 5.6–45.0) in the MPRAGE arm, with no relevant difference between study arms (HR 1.10 [95% CI: 0.78–1.56], log-rank *P* = .585; Figure 2B). The cause of death was documented for 108 of 138 patients (78.3%) in the overall cohort. Fourteen patients (10.1%) died from BM and 94 patients (68.1%) from extracranial causes.

### Predictive and Prognostic Factors

Variable selection for multivariable analysis was done according to the prespecified criteria elaborated above. Supplementary Table S2 shows the variables, which qualified for multivariable Cox regression for freedom from WBRTi. Having 5–10 BM at baseline predicted shorter freedom from WBRTi (HR 3.13, 95% CI: 1.53–6.40, *P* = .002). Supplementary Table S3 displays the respective variables analyzed for OS. Factors prognostic of longer OS included Karnofsky Performance Status > 80% (HR 0.51, 95% CI: 0.33–0.77, *P* = .002) and concomitant immunotherapy (HR 0.34, 95% CI: 0.23–0.52, *P* < .001) or targeted therapy (HR 0.51, 95% CI: 0.34–0.78, *P* = .002). The initial number of BM did not significantly affect OS. Patients receiving immunotherapy had a median OS (mOS) of 18.1 months versus 9.0 months without it (95% CI: 13.6–26.6 vs 7.2–11.3). Those on targeted therapy showed a mOS of 19.5 months compared to 9.6 months without (95% CI: 13.0–28.7 vs 7.5–13.0). The median OS was considerably higher, at 19.5 months, for patients on either aforementioned modern systemic treatment versus 4.4 months without (95% CI: 14.8–25.1 vs 2.9–7.2).

### Safety

Table 3 displays adverse events considered treatment-related with a probability of “possibly” or higher (TRAE). There were 108 reports of TRAE of any grade in the SPACE arm and 148 in the MPRAGE arm. Of those, 10 TRAE of grade 3 occurred in the SPACE arm and 17 in the MPRAGE arm. Most common grade 3 TRAE were radionecrosis (*n* = 9, 4.6% of overall cohort), seizures (*n* = 7, 3.6%), and focal neurological deficits (*n* = 7, 3.6%). One grade 4 seizure (status epilepticus) was observed in 1 patient in the MPRAGE arm. One patient in the SPACE arm died of a postoperative complication following neurosurgical resection of a symptomatic radionecrosis, while simultaneously suffering systemic progression. There were no directly treatment-associated deaths in either group. Patients receiving concomitant immunotherapy or targeted therapy (I/T Therapy) had no higher incidence of TRAE than patients without. The exposure-adjusted incidence rate (EIAR) of any grade TRAE, calculated by normalizing the average incidence rate per patient by the time on trial, was 1.285 in patients with I/T Therapy versus 1.970 in patients without (Supplementary Table S4).

**Table 3.** Adverse Events Rated Treatment-Related With a Probability of “Possibly” or Higher (TRAE)

	SPACE (*n* = 93)			MPRAGE (*n* = 99)		
	Any grade	Grade 3	Grade 4/5	Any grade	Grade 3	Grade 4/5
Any	108 (total)	10 (total)	1 (total)	148 (total)	17 (total)	1 (total)
Headaches	18 (19%)	0 (0%)	—	22 (22%)	0 (0%)	—
Nausea	3 (3%)	0 (0%)	—	5 (5%)	0 (0%)	—
Seizures	10 (11%)	2 (2%)	—	9 (9%)	5 (5%)	1 (1%)
Focal neurological deficits	15 (16%)	3 (3%)	—	33 (33%)	4 (4%)	—
Radiation dermatitis	1 (1%)	1 (1%)	—	1 (1%)	0 (0%)	—
Focal hair loss	11 (12%)	0 (0%)	—	10 (10%)	0 (0%)	—
Fatigue	46 (49%)	1 (1%)	—	59 (60%)	2 (2%)	—
Radionecrosis	4 (4%)	3 (3%)	1 (1%)	14 (14%)	6 (6%)	—

Data are numbers (%), unless otherwise speciﬁed.

## Discussion

In this prospective randomized phase 2 trial, SRS for multiple BM followed by close monitoring and repeated SRS for new lesions avoided the need for WBRT and prevented neurologic death in a large majority of patients. Only one-fifth of patients reached the per-protocol indication for salvage WBRT within 2 years. Upfront and repeated SRS for up to 10 simultaneous lesions was well-tolerated. Simultaneous combination with individually indicated standard-of-care systemic treatment, including immunotherapy and targeted therapies, did not yield any safety signals and was associated with favorable OS, as was good clinical performance. A higher initial number of BM predicted shorter freedom from WBRTi, but not OS.

National Comprehensive Cancer Network (NCCN) guidelines recommend SRS for patients with limited BM and good performance status. For patients with multiple BM, WBRT, with or without hippocampal avoidance (HA-WBRT), is primarily recommended.^19^ Still, HA-WBRT, even combined with memantine to mitigate neurocognitive decline, carries a high risk, with over 50% of patients experiencing decline within 6 months. Thus, SRS for multiple BM could present a viable alternative to mitigate this risk and potentially circumvent the need for WBRT.

The most comprehensive evidence on SRS for multiple BM comes from the JLGK0901 prospective cohort study by Yamamoto et al., which showed comparable survival in patients with 2–4 and 5–10 BM treated with SRS.^20^ They also reported similar salvage WBRT rates of 8%–11% across different lesion counts. The CROSS-FIRE study, a large multicenter cohort study by Rusthoven et al., used the JLGK0901 patients for comparison against retrospective data from over 4700 patients with NSCLC BM and 892 with SCLC BM, all treated initially with SRS. This study observed a salvage WBRT rate of 10.9% in the NSCLC subgroup.^21^ A large, retrospective multicenter analysis of 2089 patients by Hughes et al. described a 2-year salvage WBRT rate of 14% and salvage SRS rate of 20% without significant differences between patients with 1, 2–4, or 5–15 BM.^22^ A prospective single-arm study by Nichol et al., found a salvage WBRT rate of 12% in patients with imaging follow-up, though the incidence of intracranial recurrence was upwards of 30% at 2 years and not all patients were offered salvage treatment.^23^ Additionally, 2 retrospective cohort studies involving 300 patients with NSCLC BM treated with upfront and repeated SRS found salvage WBRT rates of 19.3% and 20%, respectively.^17,24^

Our trial differs from the aforementioned data in several relevant aspects. Given that rigorous MRI follow-up and re-treatment for new lesions were integral to our study protocol, the information we gathered on patient outcomes post-initial SRS is arguably more comprehensive than in other studies. In the JLGK0901 study, MRI follow-up was not mandated and unavailable for 10% of patients.^7^ In the retrospective studies discussed previously, the availability of systematic MRI follow-up is even more uncertain, thus an underestimation of the incidence of new BM and salvage WBRT must be assumed. On the other hand, in our study the 3-monthly regularity of high-sensitivity MRI possibly resulted in an increase in the detection of new metastases, and in consequence, to a higher rate of patients requiring repeated SRS or ultimately reaching WBRTi according to protocol definition. Yet, even with this intensive monitoring, our salvage WBRTi rate of 20.9% (crude rate at 24 months) is notably low. To quantify our primary outcome, we chose a precise and observer-independent definition of WBRTi as a surrogate for patients requiring salvage WBRT. This was defined as the simultaneous new occurrence or progression of >10 BM or occurrence of LMD. In current clinical practice, this situation might not always lead to WBRT, thanks to enhanced SRS capabilities and the availability of intracranially active systemic agents. However, at the time of trial design, forgoing WBRT for patients with over 10 active BM was not substantiated by strong evidence, and options for intracranially active treatments were limited. Importantly, reaching WBRTi in our study did not automatically result in WBRT. As detailed in Table 2, a third of the patients reaching WBRTi opted for alternative interventions like systemic treatment (*n* = 4, 2.1%), additional rounds of SRS (*n* = 1, 0.5%), or best supportive care (*n* = 6, 3.1%) instead of WBRT. In consequence, the actual rate of salvage WBRT at 15.1% was lower than indicated by our protocol-based analysis.

In our study, the median OS of 13.1 months was favorable compared to other patient collectives with multiple BM and histology as well as extracerebral tumor status at baseline were not predictive for OS. The JLGK0901 trial reported mOS of 10.8 months for patients with 2 or more tumors, and 13.9 months for those with a single tumor.^7^ The retrospective multicenter analysis by Hughes et al. found a mOS of 11.2 months with significant differences between patients with 1 BM (mOS 14.6 months), 2–4 BM (mOS 9.5 months), and 5–15 BM (mOS 7.5 months).^22^ Several other analyses reported mOS durations between 6.8 and 10.4 months.^20,23,25^ Lastly, the CROSS-FIRE cohort study found a mOS of 10.5 months in patients with NSCLC and BM treated with upfront SRS.^21^ In our study, the mOS was notably higher for patients receiving immunotherapy or targeted therapies: 18.1 months with immunotherapy and 19.5 months with targeted therapy. This illustrates that the concurrent use of modern systemic treatments alongside SRS contributed to the favorable OS. In line with our findings, the TURBO-NSCLC study, among similar analyses, described improved OS in patients with oncogenic-driven NSCLC, when modern targeted therapies were combined with upfront SRS.^26–28^ Similarly, combining immunotherapy with SRS can contribute to a longer time to distant intracranial failure in selected histologies.^29,30^ However, these studies are retrospective and do not specifically explore the safety of the concurrent approach. This highlights our study’s unique position as a contemporary, prospective trial that systematically integrated these treatments concomitant to SRS. Importantly, we observed no safety concerns or increase in treatment-related adverse events with this combination. In fact, the exposure-adjusted incidence rate of TRAE was lower in the subgroup receiving concomitant I/T Therapy versus those without, suggesting this combined approach is safe and potentially enhances survival.

In our trial, the use of upfront and salvage SRS for recurring multiple BM led to a low 10% rate of neurologic death, indicating a minimal impact of BM on overall prognosis. Our results compare favorably to rates reported previously: historical neurologic death rates before the introduction of SRS were around 35%–37%.^3,31^ More recent studies, including SRS have reported rates of 17%–36%, depending on histology.^23,32,33^ In the JLGK0901 trial, the neurologic death rate was fairly low at <10%. This emphasizes the role of close monitoring and salvage treatment, though in JLGK0901, this was not explicitly part of the study protocol, as it was in our trial. Also, in JLGK0901, imaging follow-up was missing for 10% of patients, mostly those with early deaths or clinical deterioration soon after initial SRS, which possibly led to an underestimation of neurologic death rates.^7^ Our results suggest that proactive management of BM and immediate salvage SRS for new lesions can further reduce neurologic death risk, supporting previous findings of better prognosis when lesions were treated at an earlier stage and smaller volume.^34^

We observed no significant differences in freedom from WBRTi or OS based on lesion detection and treatment with either SPACE or MPRAGE sequences. A previous diagnostic study by Kato et al. highlighted SPACE’s superior sensitivity and specificity, especially for detecting small BM, compared to MPRAGE, which has later been confirmed in the context of SRS.^13,14^ This difference may be attributed to SPACE’s higher contrast enhancement, being a spin-echo sequence, compared to MPRAGE, which is a gradient echo sequence. Additionally, SPACE suppresses the intensity of white matter and cortical vessels more strongly than MPRAGE (“black blood effect”).^12,13^ Welzel et al. confirmed these findings, reporting that post-gadolinium SPACE detected 29.8% more BM than post-gadolinium MPRAGE and 16.9% more than post-gadolinium late-phase MPRAGE in a study of 199 patients planned for SRS.^15^

However, despite these advantages of SPACE, it did not significantly affect clinical outcomes in our trial. Notably, the rate of WBRTi in the control MPRAGE arm was lower than initially expected, contributing to this result. This may be because MPRAGE, although less sensitive, is still highly effective in detecting small BM. In our study, both sequences were three-dimensionally acquired with multiplanar reconstructions and a 1 mm slice thickness, enabling the detection of BM smaller than 3 mm. Accordingly, in both study arms, 3-monthly scans with proactive management of new lesions effectively prevented intracranial dissemination, WBRT indication, and neurological death, irrespective of the sequence used. The immediate re-treatment for new lesions in both arms, paired with defining WBRTi based on new/untreated lesions (as opposed to the total count of lesions), possibly contributed to reducing the impact of SPACE on the incidence of WBRTi in the long term, compared to MPRAGE. On the other hand, in the short term, treatment based on the more sensitive SPACE sequence has been demonstrated to prolong time to first distant brain failure.^14^ In a resource-constrained environment with limited capacities for repeated MRI, this could provide practical advantages: detecting occult lesions early and thus treating patients more comprehensively upfront could possibly permit less frequent scans during follow-up and reduce the need for frequent re-treatment.^14^ The absence of clinical outcome advantage of the more sensitive MRI sequence has further practical implications as MPRAGE and similar gradient echo sequences are more widely established and available than SPACE or comparable 3D Turbo Spin Echo (TSE) sequences. Our trial underscores the importance of consistent, high-resolution MRI monitoring and proactive lesion management over the choice of a specific imaging sequence.

Ideally, a trial randomizing SRS against the current standard of WBRT or HA-WBRT with the endpoint of OS would be most rigorous for establishing SRS for multiple BM. Ongoing trials of a similar design include the phase 3 CCTG CE.7 trial (NCT03550391) for patients with 5–15 BM, the phase 2 CyberChallenge trial (NCT05378633) for 4–15 BM and the phase 3 SHARP trial (NCT06457906) for up to 10 BM. The phase 3 HipSter trial (NCT04277403) also compares HA-WBRT and SRS for 4–15 BM, albeit with the primary endpoint of intracranial progression-free survival. However, challenges to such trials include slow patient accrual, as shown by the premature termination of the NCT01592968 and NCT02353000 trials.^35,36^ These difficulties arise partly because some providers are already offering patients SRS for multiple BM, based on the convincing evidence for the deleterious impact of WBRT on QoL. We chose freedom from WBRTi as clinically meaningful primary endpoint for several reasons: While overall intracranial control is inferior following SRS due to distant brain recurrences, these can be effectively managed, as our results demonstrate. Overall survival on the other hand is relevantly influenced by extracranial disease and therefore also not an optimal endpoint. Lastly, the strategy of upfront SRS for multiple BM may in some cases still require WBRT as salvage treatment at a later stage. Therefore, this crossover could confound the results of a trial randomizing between SRS and WBRT.

Our study’s primary limitation is its single-center design, with all patients treated at a CyberKnife center and by personnel highly experienced in intracranial SRS. Despite this, the growing use of conventional linacs equipped for stereotactic treatments and SRS-specific software makes our findings relevant to the wider oncologic community. Comparable dosimetric plan quality reported with linac-based solutions supports this applicability.^8–10^

We aimed to minimize selection bias by setting broad inclusion criteria. Our high recruitment rate averaging 1.7 patients per week at a single center suggests successful broad inclusion, reducing selection bias. Stratification variables were chosen for their previously described effect on survival and to avoid imbalances in survival, death from extracranial causes being the most important competing risk.^7^ We did not stratify for number of lesions and extracerebral disease status, however, these factors were very well balanced between arms (Table 1). While our study did not restrict inclusion to specific histologies, randomization was stratified to avoid imbalances in histology between study arms. However, our inclusive approach led to small subgroups for rarer histologies, limiting the possibility of representative subgroup analyses.

## Conclusion

In conclusion, the CYBER-SPACE trial provides strong evidence supporting the use of SRS for treating up to ten simultaneous BM in adult cancer patients. In these patients, SRS with consecutive thorough monitoring and immediate re-treatment for new lesions may effectively decrease the need for WBRT and result in a low rate of neurologic death. In a setting, where monitoring and immediate re-treatment are performed consistently, a more sensitive method of MRI compared to routine MPRAGE did not contribute to more improved outcome in the long term. The concurrent use of SRS with novel systemic treatments was well-tolerated, showing no increase in treatment-related adverse events. Based on our results, SRS should be considered a favorable alternative to WBRT for patients with up to 10 BM.

## Supplementary material

Supplementary material is available online at *Neuro-Oncology* (https://academic.oup.com/neuro-oncology).

noae201_suppl_Supplementary_Figures_S1-S2_Tables_S1-S4

## Data sharing

De-identified patient data from this study along with a data dictionary can be made available after a signed data access agreement upon reasonable request to the corresponding author.
