# Supplementary material for: Stereotactic radiosurgery for 1–10 brain metastases to avoid whole-brain radiotherapy: Results of the CYBER-SPACE randomized phase 2 trial
Source: Neuro Oncol. 2024 Sep 28;27(2):479–91. doi: 10.1093/neuonc/noae201 (PMC11812257; doi:10.1093/neuonc/noae201)
Supplement: noae201_suppl_Supplementary_Figures_S1-S2_Tables_S1-S4 [file noae201_suppl_supplementary_figures_s1-s2_tables_s1-s4.docx]

Supplementary Material

# Supplementary Tables

Supplementary Table 1 Exclusions from the mITT dataset and reasons for exclusion. These patients were excluded from the trial before starting study treatment and were consequently not treated or followed up according to study protocol. These exclusions were independent from the randomization result. Therefore, removing these patients from all analyses is not expected to lead to bias.

| **Study group** | **Subject ID** | **Reason for exclusion** |
| --- | --- | --- |
| SPACE | 7 | Death from reasons unrelated to BM before initiation of study treatment |
| MPRAGE | 12 | Randomized mistakenly despite violating inclusion/exclusion criteria: >10 metastases in screening MRI |
| SPACE | 54 | Randomized mistakenly despite violating inclusion/exclusion criteria: Radiographic evidence of LMD in screening MRI |
| SPACE | 94 | Consent withdrawn after randomization and before beginning of treatment |
| MPRAGE | 123 | Death from reasons unrelated to BM before initiation of study treatment |
| SPACE | 124 | Randomized mistakenly despite violating inclusion/exclusion criteria: >10 metastases in screening MRI |
| SPACE | 143 | Randomized mistakenly despite violating inclusion/exclusion criteria: No evidence of previously reported cerebral metastasis in screening MRI |
| MPRAGE | 183 | Death from reasons unrelated to BM before initiation of study treatment |
| SPACE | 196 | Death from reasons unrelated to BM before initiation of study treatment |
| MPRAGE | 199 | Randomized mistakenly despite violating inclusion/exclusion criteria: >10 metastases in screening MRI |

Supplementary Table 2 Clinical predictive factors* for freedom from whole-brain radiotherapy in multivariable analysis.

|  | **Hazard Ratio*** | **95%-CI** | **p-value** |
| --- | --- | --- | --- |
| Treatment allocation: SPACE | 0.98 | 0.52 – 1.86 | 0.9454 |
| Concomitant Immunotherapy | 0.56 | 0.28 - 1.09 | 0.0881 |
| Concomitant targeted therapy | 1.23 | 0.62 - 2.43 | 0.5599 |
| Concomitant other therapy | 0.52 | 0.11 - 2.39 | 0.4002 |
| Synchronous BM | 1.63 | 0.81 - 3.28 | 0.1721 |
| Tumor extracerebrally controlled | 0.96 | 0.45 - 2.04 | 0.9030 |
| **5-10 BM in initial MRI** | **3.13** | 1.53 - 6.40 | **0.0018** |
| Singular BM in initial MRI | 1.04 | 0.38 - 2.86 | 0.9469 |

BM=brain metastases; CI=confidence interval; MRI=magnetic resonance imaging. *Hazard ratio adjusted for all clinical factors listed in this table. *factors analyzed in univariate analysis: age, sex, histology, concomitant therapy, time of appearance of brain metastases, location of brain metastases, largest diameter of lesions, volume of largest brain metastasis at baseline, presence of extracerebral metastases, extracerebral tumor status, total number of lesions at baseline, Karnofsky Performance Status, RPA class, GPA score, dsGPA score, HVLT-R: Total recall score at baseline, prescription dose of SRS, PDL1-Score, presence of oncogenic mutation for lung cancer (EGFR, ALK, BRAF, ROS1)

Supplementary Table 3 Clinical prognostic factors for overall survival in multivariable analysis.

|  | **Hazard Ratio*** | **95%-CI** | **p-value** |
| --- | --- | --- | --- |
| Treatment Allocation: SPACE | 1.29 | 0.90 – 1.86 | 0.1619 |
| GPA-Score: 0-1 | 0.86 | 0.55 – 1.35 | 0.5136 |
| GPA-Score: 3 | 0.54 | 0.25 – 1.17 | 0.1206 |
| GPA-Score: 3.5-4 | 1.26 | 0.29 – 5.53 | 0.7571 |
| **KPS >80%** | **0.51** | 0.33 - 0.77 | **0.0015** |
| Tumor extracerebrally controlled | 0.70 | 0.45 - 1.11 | 0.1268 |
| **Concomitant targeted therapy** | **0.51** | 0.34 - 0.78 | **0.0019** |
| **Concomitant immunotherapy** | **0.34** | 0.23 - 0.52 | **<.0001** |
| Sex | 0.72 | 0.49 - 1.06 | 0.0943 |
| HVLT-R: Total recall score | 0.98 | 0.95 – 1.01 | 0.1352 |

BM=brain metastases; CI=confidence interval; KPS=Karnofsky performance status; HVLT-R=Hopkins verbal learning test-revised at baseline. *Hazard ratio adjusted for all clinical factors listed in this table.

Supplementary Table 4 Comparison of adverse events rated treatment-related with a probability of “possibly” or higher (TRAE) between patients receiving concomitant immuno- or targeted therapies (I/T Therapy) versus all others. Exposure-adjusted incidence rates (EAIR) per patient have been calculated by normalizing the average incidence rate per patient by the time on trial to account for differences in survival between the subgroups and provide a more meaningful comparison.

|  | **I/T Therapy (135 patients)** | | | **no I/T Therapy (57 patients)** | | |
| --- | --- | --- | --- | --- | --- | --- |
| **CTCAE** | **absolute frequency** | **average incidence per patient** | **EAIR** | **absolute frequency** | **average incidence per patient** | **EAIR** |
| Grade 1 | 161 | 1.193 | 0.838 | 35 | 0.614 | 1.060 |
| Grade 2 | 61 | 0.452 | 0.317 | 20 | 0.351 | 0.601 |
| Grade 3 | 23 | 0.170 | 0.120 | 10 | 0.175 | 0.303 |
| Grade 4 | 1 | 0.007 | 0.005 | 0 | 0 | 0 |
| Grade 5 | 1 | 0.007 | 0.005 | 0 | 0 | 0 |
| Any grade | 247 | 1.830 | 1.285 | 65 | 1.140 | 1.970 |

I/T Therapy=immunotherapy or targeted therapy; CTCAE=Common Terminology Criteria for Adverse Events, EIAR=Exposure adjusted incidence rate.

# Supplementary Figures


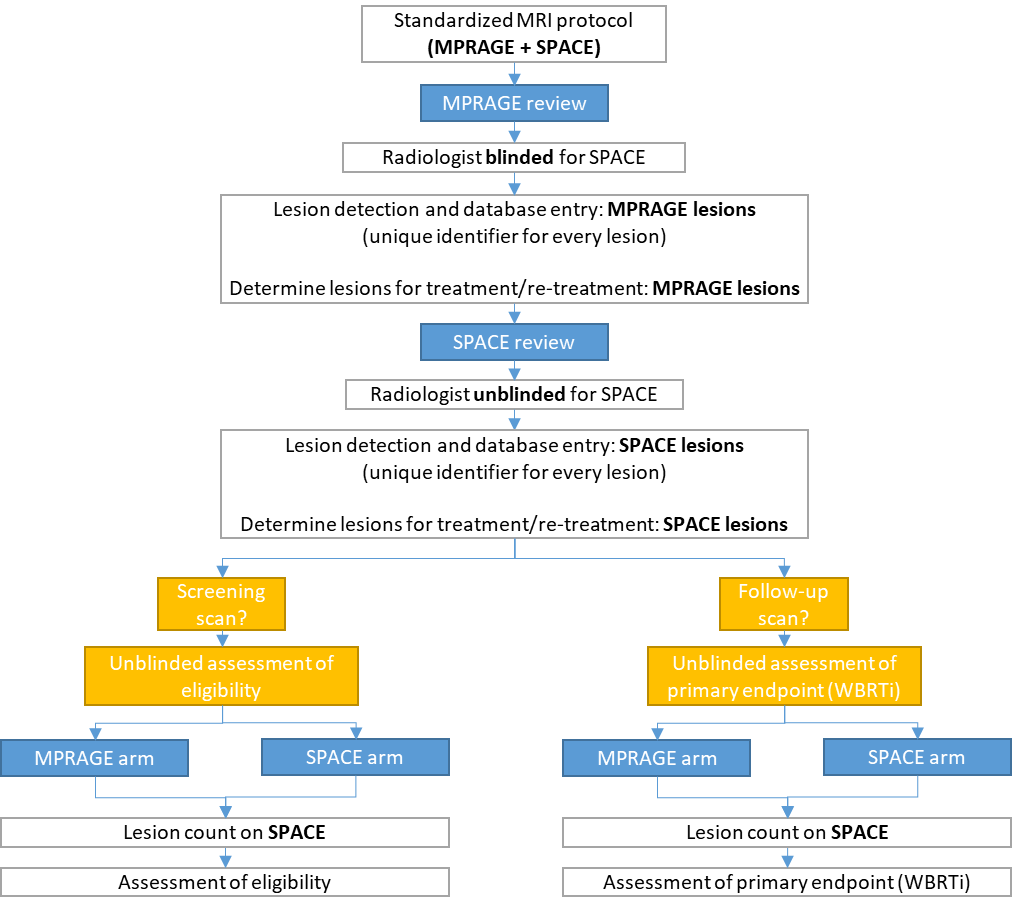


Supplementary Figure 1 Standardized workflow for central image review by the study radiologist. The standardized MRI protocol encompassed both MPRAGE and SPACE sequences for all study patients. For initial MPRAGE sequence review and lesion detection/lesion tracking, the radiologist was blinded to the more sensitive SPACE sequence. Individual lesions were then defined and entered into a database using a dedicated nomenclature and unique identifier for the purpose of longitudinal tracking for every lesion, detailing the series/image information within the scan. After database entry for MPRAGE lesions, the same radiologist was then unblinded for SPACE and entered all lesions visible in SPACE into the database. For evaluation of study eligibility (screening MRI) or the primary endpoint of WBRTi (follow-up MRI), lesions were counted on the SPACE sequence in both arms (MPRAGE and SPACE). This avoids potential imbalances regarding number of metastases at study inclusion, as well as imbalances in the timepoint of detection of WBRTi due to the higher sensitivity of the SPACE sequence. For initial study treatment and all re-treatments in the different arms, only lesions detected by the respective sequence (i.e. MPRAGE for MPRAGE-arm and SPACE for SPACE-arm) were treated.


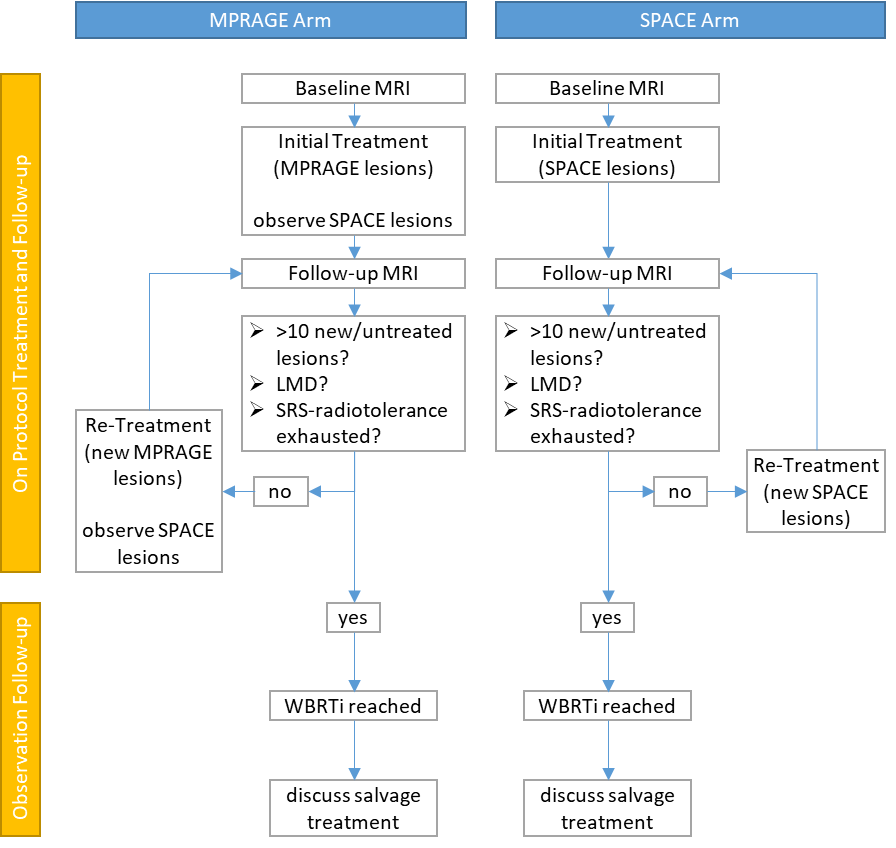


Supplementary Figure 2 Decision algorithm for assessment of the primary endpoint of “indication for whole-brain radiotherapy” (WBRTi) during follow-up. At every follow-up, only untreated/new lesions count towards the primary endpoint. Thus cumulatively, a patient could be treated for more than 10 BM before reaching WBRTi. However, only a maximum of 10 new lesions could appear simultaneously (i.e. in one follow-up scan) and be treated during one subsequent course of SRS. If more than 10 BM appeared simultaneously, this event triggered WBRTi. The selection of lesions for treatment and re-treatment was dependent on the study arm: In the SPACE arm, all lesions including those only visible in SPACE were treated. In the MPRAGE arm, only lesions visible in MPRAGE were treated and lesions only visible in SPACE were observed. If those lesions subsequently progressed, becoming visible in MPRAGE, they were then regarded as new MPRAGE lesions and treated at that later timepoint. Abbreviations: LMD – Leptomeningeal disease; SRS – Stereotactic radiosurgery.
